# Supplementary material for: Structure-guided analysis and prediction of human E2–E3 ligase pairing specificity
Source: bioRxiv. 2026 Feb 12:2026.02.10.700855. Preprint. [Version 1] doi: 10.64898/2026.02.10.700855 (PMC12919000; doi:10.64898/2026.02.10.700855)
Supplement: 4 [file NIHPP2026.02.10.700855v1-supplement-4.pdf]

bioRxiv preprint doi: https://doi.org/10.64898/2026.02.10.700855; this version posted February 12, 2026. The copyright holder for this preprint (which was not certified by peer review) is the author/funder, who has granted bioRxiv a license to display the preprint in perpetuity. It is made available under aCC-BY 4.0 International license.

Supplementary Information Appendix

Structure-guided analysis and prediction of human E2–E3 ligase pairing specificity

Brianna Jarboe[1,2], Roland L. Dunbrack, Jr.[2,*]

[1] Drexel University College of Medicine, 2900 W. Queen Lane, Philadelphia, PA 19129, USA.

[2] Institute for Cancer Research, Fox Chase Cancer Center, Philadelphia, PA 19111, USA.

*Corresponding author: roland.dunbrack@fccc.edu

bioRxiv preprint doi: https://doi.org/10.64898/2026.02.10.700855; this version posted February 12, 2026. The copyright holder for this preprint (which was not certified by peer review) is the author/funder, who has granted bioRxiv a license to display the preprint in perpetuity. It is made available under aCC-BY 4.0 International license.

Supplementary Tables

Table S1. Classification of proteins from culled E3 ligase lists

E3 Ligase Type	Count	Percentage (%)
**RING**	318	54.83%
**HECT**	28	4.83%
**RBR**	14	2.41%
**U-box**	8	1.38%
**Atypical/Hybrid**	1	0.17%
**No E3 Catalytic Domain Detected**	211	36.38%

Atypical/Hybrid: E3 ligase *G2E3* has both HECT and RING domains

bioRxiv preprint doi: https://doi.org/10.64898/2026.02.10.700855; this version posted February 12, 2026. The copyright holder for this preprint (which was not certified by peer review) is the author/funder, who has granted bioRxiv a license to display the preprint in perpetuity. It is made available under aCC-BY 4.0 International license.

Table S2. Summary of experimental structure availability by E3 ligase type

	With Structure	Without Structure	Total
E2	32	5	37
E3 HECT	21	7	28
E3 RBR	8	6	14
E3 RING	154	164	318
E3 U-box	6	2	8
E3 atypical	0	1	1
Total	221	185	406

E3 atypical: Atypical E3 ligase *G2E3* has both HECT and RING domains

PDB query date: November 10, 2025

bioRxiv preprint doi: https://doi.org/10.64898/2026.02.10.700855; this version posted February 12, 2026. The copyright holder for this preprint (which was not certified by peer review) is the author/funder, who has granted bioRxiv a license to display the preprint in perpetuity. It is made available under aCC-BY 4.0 International license.

Table S3. Summary of unique E2s and E3s in ternary complex experimental structures by type

	In Ternary	Not in Ternary	Total
E2	11	26	37
E3 HECT	1	27	28
E3 RBR	5	9	14
E3 RING	23	295	318
E3 U-box	0	8	8
E3 atypical	0	1	1
Total	40	366	406

Total ternary complex containing experimental structures, n=57

PDB query date: November 10, 2025

bioRxiv preprint doi: https://doi.org/10.64898/2026.02.10.700855; this version posted February 12, 2026. The copyright holder for this preprint (which was not certified by peer review) is the author/funder, who has granted bioRxiv a license to display the preprint in perpetuity. It is made available under aCC-BY 4.0 International license.

Table S4. Retrieved E2 genes (HGNC Gene group: Ubiquitin conjugating enzymes E2 (UBE2))

HGNC ID	HGNC Approved name	Gene Name	UniProtID	Notes
**HGNC:16710**	AKT interacting protein	*AKTIP*	Q9H8T0	non-E2 excluded from analysis
**HGNC:13516**	baculoviral IAP repeat containing 6	*BIRC6*	Q9NR09	atypical E2/E3 hybrid excluded from analysis
**HGNC:1734**	cell division cycle 34, ubq conjugating enzyme	*CDC34*	P49427	
**HGNC:12472**	ubq conjugating enzyme E2 A	*UBE2A*	P49459	
**HGNC:12473**	ubq conjugating enzyme E2 B	*UBE2B*	P63146	
**HGNC:15937**	ubq conjugating enzyme E2 C	*UBE2C*	O00762	
**HGNC:12474**	ubq conjugating enzyme E2 D1	*UBE2D1*	P51668	
**HGNC:12475**	ubq conjugating enzyme E2 D2	*UBE2D2*	P62837	
**HGNC:12476**	ubq conjugating enzyme E2 D3	*UBE2D3*	P61077	
**HGNC:21647**	ubq conjugating enzyme E2 D4	*UBE2D4*	Q9Y2X8	
**HGNC:12477**	ubq conjugating enzyme E2 E1	*UBE2E1*	P51965	
**HGNC:12478**	ubq conjugating enzyme E2 E2	*UBE2E2*	Q96LR5	
**HGNC:12479**	ubq conjugating enzyme E2 E3	*UBE2E3*	Q969T4	
**HGNC:12480**	ubq conjugating enzyme E2 F (putative)	*UBE2F*	Q969M7	
**HGNC:12482**	ubq conjugating enzyme E2 G1	*UBE2G1*	P62253	
**HGNC:12483**	ubq conjugating enzyme E2 G2	*UBE2G2*	P60604	
**HGNC:12484**	ubq conjugating enzyme E2 H	*UBE2H*	P62256	
**HGNC:12485**	ubq conjugating enzyme E2 I	*UBE2I*	P63279	
**HGNC:17598**	ubq conjugating enzyme E2 J1	*UBE2J1*	Q9Y385	
**HGNC:19268**	ubq conjugating enzyme E2 J2	*UBE2J2*	Q8N2K1	
**HGNC:4914**	ubq conjugating enzyme E2 K	*UBE2K*	P61086	
**HGNC:12488**	ubq conjugating enzyme E2 L3	*UBE2L3*	P68036	
**HGNC:13477**	ubq conjugating enzyme E2 L5	*UBE2L5*	A0A1B0GUS4	
**HGNC:12490**	ubq conjugating enzyme E2 L6	*UBE2L6*	O14933	
**HGNC:12491**	ubq conjugating enzyme E2 M	*UBE2M*	P61081	
**HGNC:12492**	ubq conjugating enzyme E2 N	*UBE2N*	P61088	
**HGNC:29554**	ubq conjugating enzyme E2 O	*UBE2O*	Q9C0C9	
**HGNC:15698**	ubq conjugating enzyme E2 Q1	*UBE2Q1*	Q7Z7E8	
**HGNC:19248**	ubq conjugating enzyme E2 Q2	*UBE2Q2*	Q8WVN8	
**HGNC:37269**	ubq conjugating enzyme E2 QL1	*UBE2QL1*	A1L167	
**HGNC:19907**	ubq conjugating enzyme E2 R2	*UBE2R2*	Q712K3	
**HGNC:17895**	ubq conjugating enzyme E2 S	*UBE2S*	Q16763	
**HGNC:25009**	ubq conjugating enzyme E2 T	*UBE2T*	Q9NPD8	
**HGNC:28559**	ubq conjugating enzyme E2 U	*UBE2U*	Q5VVX9	
**HGNC:12494**	ubq conjugating enzyme E2 V1	*UBE2V1*	Q13404	
**HGNC:12495**	ubq conjugating enzyme E2 V2	*UBE2V2*	Q15819	
**HGNC:25616**	ubq conjugating enzyme E2 W	*UBE2W*	Q96B02	
**HGNC:25847**	ubq conjugating enzyme E2 Z	*UBE2Z*	Q9H832	
**HGNC:32199**	ubq conjugating enzyme E2 E4 pseudogene	*UBE2E4P*	N/A	pseudogene excluded from analysis
**HGNC:12486**	ubq conjugating enzyme E2 L1 (pseudogene)	*UBE2L1*	N/A	pseudogene excluded from analysis
**HGNC:12487**	ubq conjugating enzyme E2 L2 (pseudogene)	*UBE2L2*	N/A	pseudogene excluded from analysis
**HGNC:12489**	ubq conjugating enzyme E2 L4 (pseudogene)	*UBE2L4*	N/A	pseudogene excluded from analysis
**HGNC:31710**	ubq conjugating enzyme E2 N like (gene/pseudogene)	*UBE2NL*	N/A	pseudogene excluded from analysis

Ubq = ubiquitin; N/A = not available

bioRxiv preprint doi: https://doi.org/10.64898/2026.02.10.700855; this version posted February 12, 2026. The copyright holder for this preprint (which was not certified by peer review) is the author/funder, who has granted bioRxiv a license to display the preprint in perpetuity. It is made available under aCC-BY 4.0 International license.

Supplementary Figures

A

Conformation/E3-type	PDB ID	PDB Structure Resolution (Å)	Interface Residues Count	UB-E2 RMSD (Å)	UB-E3 RMSD (Å)	E2-E3 RMSD (Å)	Interface RMSD (Å)
Closed/E3RING	8GRM	3.05	43	3.99	3.01	5.76	3.92
	8PJN	3.40	40	0.81	0.84	1.95	1.14

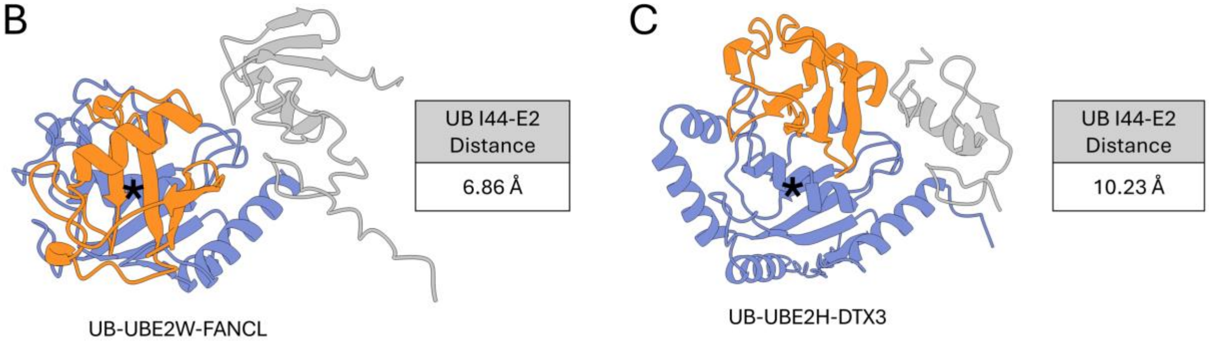

Fig. S1: AF3 models of ubiquitin–E2–E3$_{RING}$ ternary complexes without existing experimental structures with Closed/Open conformation assessment. (*A*) Table of interface RMSD values calculated by comparing experimentally determined structures of ubiquitin–E2–E3 complexes (PDB IDs listed) with their corresponding AF3-predicted models. In these AF3 predictions, the partner E3$_{RING}$ of the heterodimers present in the experimental structures were explicitly included in the model alongside the core ubiquitin–E2–E3$_{RING}$ components of the ternary complex. For 8GRM, the E3 RNF2, in a heterodimer with the E3 BMI1, makes primary contact with the E2 of the ternary complex (UBE2D2). For 8PJN, the E3 RMND5A, in a heterodimer with the E3 MAEA, makes primary contact with the E2 of the ternary complex (UBE2H). The table includes the resolution of the experimental structures, and the number of interface residues used in the RMSD calculations, defined as residues with Cα atoms within 8 Å across inter-protein interfaces of the complex. All experimental structures were released to the PDB after AF3 training dataset date cutoff of Sept. 30, 2021 (*B*) AF3 predicted structure of ubiquitin in complex with E2, UBE2W and E3, FANCL, a putative functional E2–E3 pair. This ternary complex is in the Closed conformation, based on determined 10 Å Ub Ile44-crossover helix distance cutoff. (*C*) AF3 predicted structure of ubiquitin in complex with E2, UBE2H and E3, DTX3, an E2 and E3 with no evidence of interaction. This ternary complex is in an Open conformation, based on determined 10 Å Ub Ile44-crossover helix distance cutoff. Structures were retrieved from the RCSB Protein Data Bank and visualized using UCSF ChimeraX version 1.10.1. Asterisk (*) indicates crossover helix of the E2 in the ternary complexes.

bioRxiv preprint doi: https://doi.org/10.64898/2026.02.10.700855; this version posted February 12, 2026. The copyright holder for this preprint (which was not certified by peer review) is the author/funder, who has granted bioRxiv a license to display the preprint in perpetuity. It is made available under aCC-BY 4.0 International license.

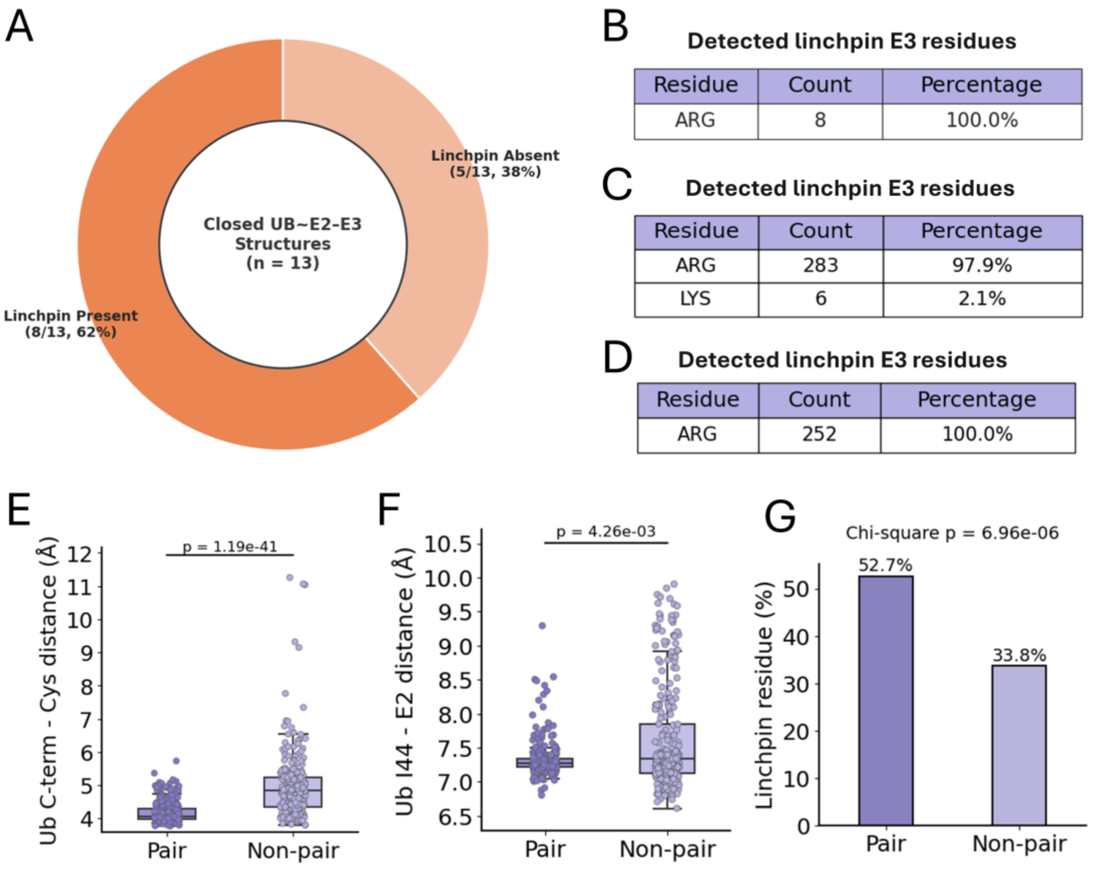

Fig. S2: Analysis of linchpin residues in experimental, AF3, and CF models ternary complexes, and additional structural features of pair and non-pair ubiquitin–E2–E3 Closed conformation ternary complex structures generated with ColabFold. (*A*) Proportion of Closed conformation experimental ubiquitin~E2–E3$_{RING}$ ternary complexes featuring an E3 ligase linchpin residue. (*B*) Table of detected E3 linchpin residue types in Closed conformation experimental ubiquitin~E2–E3$_{RING}$ ternary complexes. (*C*) Table of detected E3 linchpin residue types across AF3 modeled ternary complexes of both pair and non-pair E2–E3 sets. (*D*) Table of detected E3 linchpin residue types across CF modeled ternary complexes of both pair and non-pair E2–E3 sets. (*E)* Distances from ubiquitin Ile44 Cα to closest crossover helix Cα in CF modeled ternary complexes, p-value calculated using Mann-Whitney U Test (two-sided). (*F*) Distances from ubiquitin terminal glycine Cα to the E2 active-site cysteine carbonyl carbon Cα in CF modeled ternary complexes, p-value calculated using Mann-Whitney U Test (two-sided). (*G*) Proportion of CF modeled ternary complexes featuring linchpin residue in pair and non-pair complexes. (*E*-*G*) *n* = 298 (pair) and *n* = 281 (non-pair).

bioRxiv preprint doi: https://doi.org/10.64898/2026.02.10.700855; this version posted February 12, 2026. The copyright holder for this preprint (which was not certified by peer review) is the author/funder, who has granted bioRxiv a license to display the preprint in perpetuity. It is made available under aCC-BY 4.0 International license.

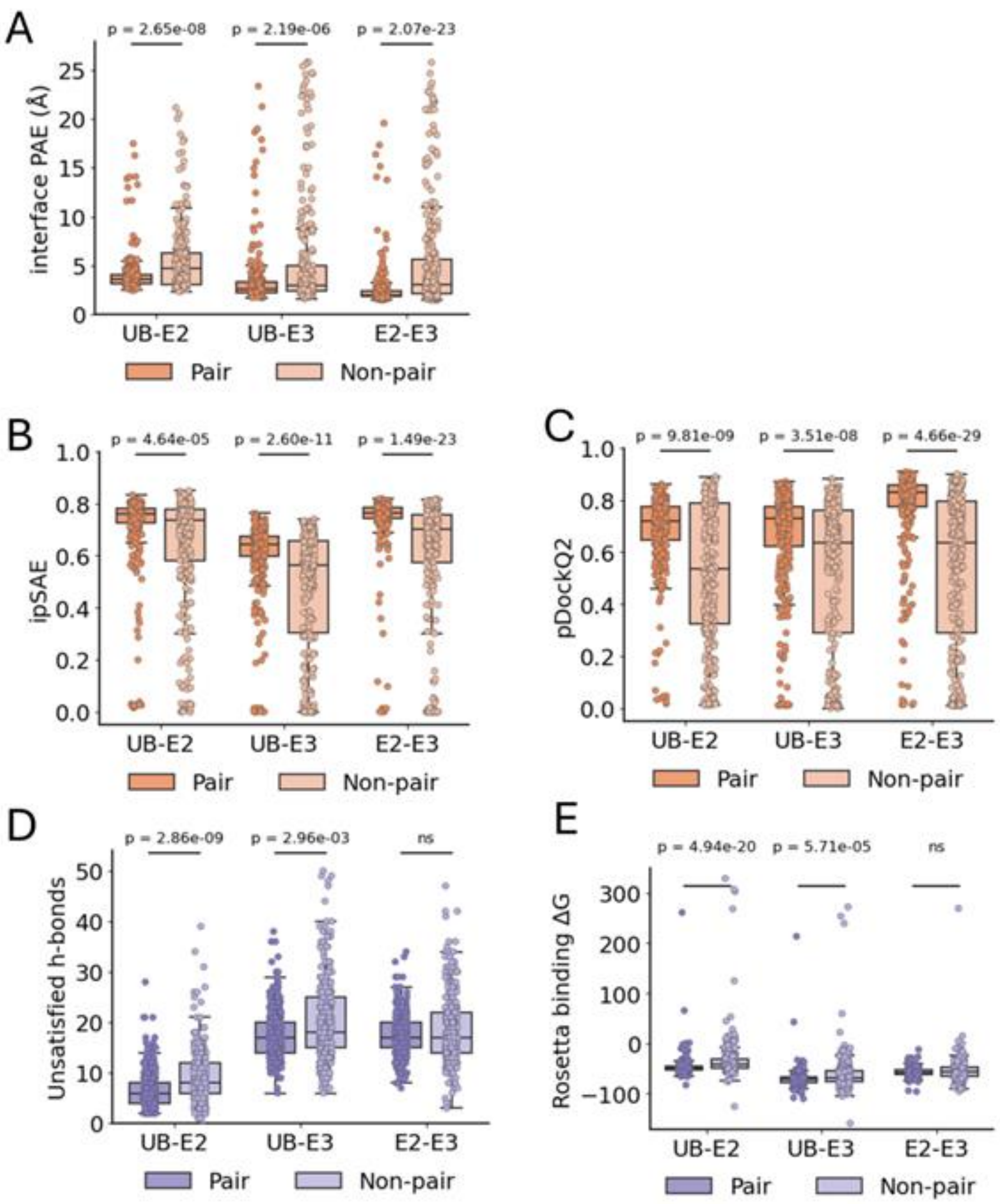

Fig. S3: ColabFold modeling of ubiquitin–E2–E3 ternary complexes of E2–E3 pair and non-pair sets with Closed/Open conformation analysis. (*A*) Scatter plot of ubiquitin Ile44 to E2 crossover helix distance vs ubiquitin Gly C-terminus to E2 catalytic cysteine distance for all structures. (Note: Structures containing the E2s UBE2V1 or UBE2V2, which lack a catalytic cysteine, are not shown in this plot. However, all such structures exceeded the 10 Å I44–E2 crossover helix distance cutoff and were assigned Open conformation designation.) (*B*) Proportion of ternary complexes in a Closed vs. Open conformation for pair and non-pair set. Closed/Open proportion by E2 (*E*), $E3_{RBR}$ (RBR-type E3) (*C*), and the 30 lowest Closed proportion ranking $E3_{RING}$ (RING/U-box-type E3) (*D*) (E2s and E3s referred to by gene name).

bioRxiv preprint doi: https://doi.org/10.64898/2026.02.10.700855; this version posted February 12, 2026. The copyright holder for this preprint (which was not certified by peer review) is the author/funder, who has granted bioRxiv a license to display the preprint in perpetuity. It is made available under aCC-BY 4.0 International license.

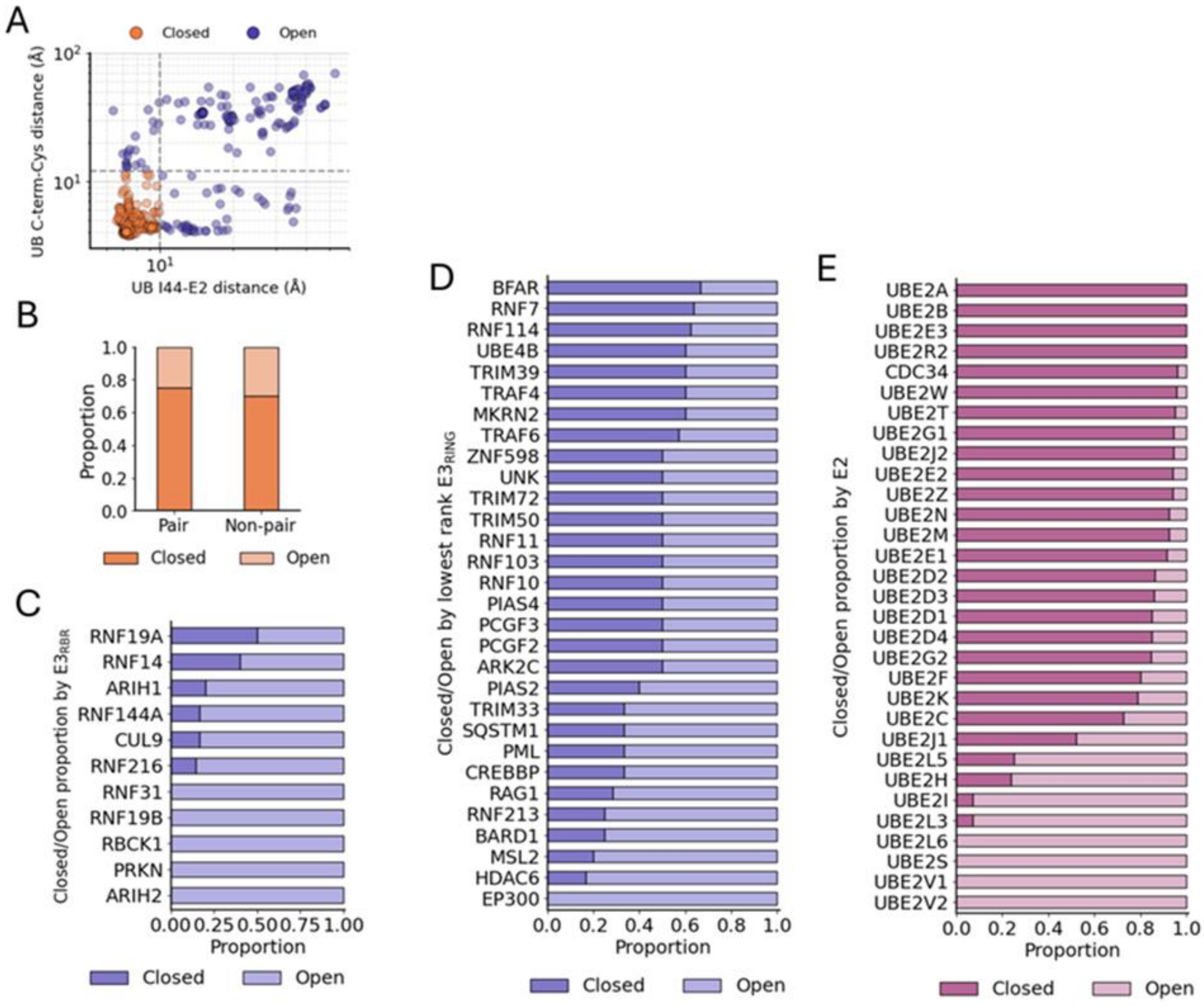

Fig. S4: Interface confidence metrics, energetics, and interactions of pair and non-pair ubiquitin–E2–E3 Closed conformation ternary complex structures generated with ColabFold. Average interface PAE values (based on 8 Å Cα-Cα interface cutoff) (*A*), ipSAE (*B*), and pDockq2 (*C*) plotted for each complex interface. Number of unsatisfied hydrogen bonds at interfaces (*D*) and complex interface binding energies (Rosetta units) (*E*), both determined using Rosetta InterfaceAnalyzer. Two-sided Mann-Whitney U tests were used to calculate p-values; ns denotes $p > 0.05$. For panels b–e, n = 298 (pair) and n = 281 (non-pair). For panel a, group sizes vary by interface (due to exclusion of structures lacking interface residues within the 8 Å cutoff): UB–E2: n = 298 (pair), n = 281 (non-pair); UB–E3: n = 298 (pair), n = 275 (non-pair); E2–E3: n = 298 (pair), n = 281 for non-pair.
